# Clear-cut observation of clearance of sustainable upconverting nanoparticles from lymphatic system of small living mice

**DOI:** 10.1038/srep27407

**Published:** 2016-06-06

**Authors:** Hye Sun Park, Sang Hwan Nam, Jongwoo Kim, Hyung Seon Shin, Yung Doug Suh, Kwan Soo Hong

**Affiliations:** 1 Bioimaging Research Team, Korea Basic Science Institute, Cheongju 28119, Korea; 2 Laboratory for Advanced Molecular Probing (LAMP), Research Center for Convergence NanoRaman Technology, Korea Research Institute of Chemical Technology, Daejeon 34114, Korea; 3 Environmental Monitoring & Research Team, Korea Basic Science Institute, Cheongju 28119, Korea; 4 School of Chemical Engineering, Sungkyunkwan University, Suwon 16419, Korea; 5 Bioanalytical Science, University of Science and Technology, Daejeon 34113, Korea; 6 Graduate School of Analytical Science and Technology, Chungnam National University, Daejeon 34134, Korea

## Abstract

The significance of lymphatic system has gathered great attention for immunotechnology related to cancer metastasis and immunotherapy. To develop innovative immunodiagnostics and immunotherapy in *in vivo* environments, it is very important to understand excretion pathways and clearance of injected cargoes. Herein, we employed Tm^3+^-doped upconverting nanoparticles (UCNPs) with versatile advantages suitable for long-term non-invasive *in vivo* optical imaging and tracking. Transport and retention of the UCNPs in the lymphatic system were evaluated with high-quality NIR-to-NIR upconversion luminescence (UCL) imaging. We obtained their kinetic luminescence profiles for the injection site and sentinel lymph node (SLN) and observed luminescence signals for one month; we also examined UCL images in SLN tissues, organs, and faeces at each time point. We speculate that the injected UCNPs in a footpad of a small mouse are transported rapidly from the lymphatic system to the blood system and then eventually result in an efficient excretion by the hepatobiliary route. These results will support development of novel techniques for SLN biopsy as well as immunotechnology.

The lymphatic system plays an important role in the diagnosis of cancer metastasis and immunotherapeutic responses in the human body^1^,^2^. Lymph nodes are major organs in lymphatic system, acting as a protector from foreign materials and as a communicating area with confluent immune cells. The state of lymph node reflects the change of immune system against inflections or diseases. Especially, it is important to identify the nearest lymph node (sentinel lymph node, SLN) to tumor tissue for diagnosis of cancer metastasis because cancer cells spread out through lymph node.

For sentinel lymph node (SLN) mapping and biopsy techniques, imaging probes containing fluorophores have been widely developed in view of accurate positioning with longer retention in SLN^3^,^4^,^5^,^6^. Organic fluorophores such as indocyanine green (ICG) have been applied in clinical use for SLN mapping and their modified nanomaterials have been studied to overcome their limits, rapid and extensive dispersion along the lymphatic flow which cause imprecise positioning of sentinel node^3^,^4^. However, they may also give misleading results due to their biodegradation in *in vivo* environments and photobleaching from repeated exposure to light. In particular, in long-term *in vivo* imaging, the probes are desired to have photostability and signal uniformity to facilitate quantitative analysis from the obtained images and low *in vivo* toxicity even in the condition of long circulation.

Recently, lanthanide-doped upconverting nanoparticles (UCNPs) have gathered great attention because of their photostability and biocompatibility^7^,^8^,^9^,^10^. UCNPs are free from autofluorescence background and thus provide a high signal-to-noise ratio (SNR). In particular, near-infrared (NIR) emission of Tm^3+^-doped UCNPs facilitates highly sensitive detection (SNR > 30) with a large penetration depth (>2 cm) in biological tissues^11^. It has been reported that *in vivo* NIR imaging using Tm^3+^-doped UCNPs could provide improved visualization in lymphatic imaging compared to UCNP-based visible or quantum dot (QD)-based fluorescence imaging^12^,^13^.

One of the most overlooked factors in the study of lymphatic imaging using nanoprobes is their clearance after assuming roles. Recently, fluorescent NIR dye doped-silica nanoparticles were employed in a study of long-term *in vivo* SLN mapping in the lymphatic system, and efficient excretion from mice was demonstrated^14^,^15^. However, long-term *in vivo* imaging and biodistribution study using UCNPs in the lymphatic system have not been reported yet.

In this study, the lymphatic excretion property of Tm^3+^-doped UCNPs was investigated in mice on a long term. Transport and retention of the nanoparticles in lymphatic mapping were evaluated with highly sensitive, high-quality images. Their kinetic luminescence profiles in SLN mapping for injection sites as well as axillary lymph nodes were characterized with *in vivo* quantitative imaging analysis. *Ex vivo* biodistribution of the UCNPs was also investigated to confirm their clearance from the lymphatic system.

## Results

**Figure 1:**
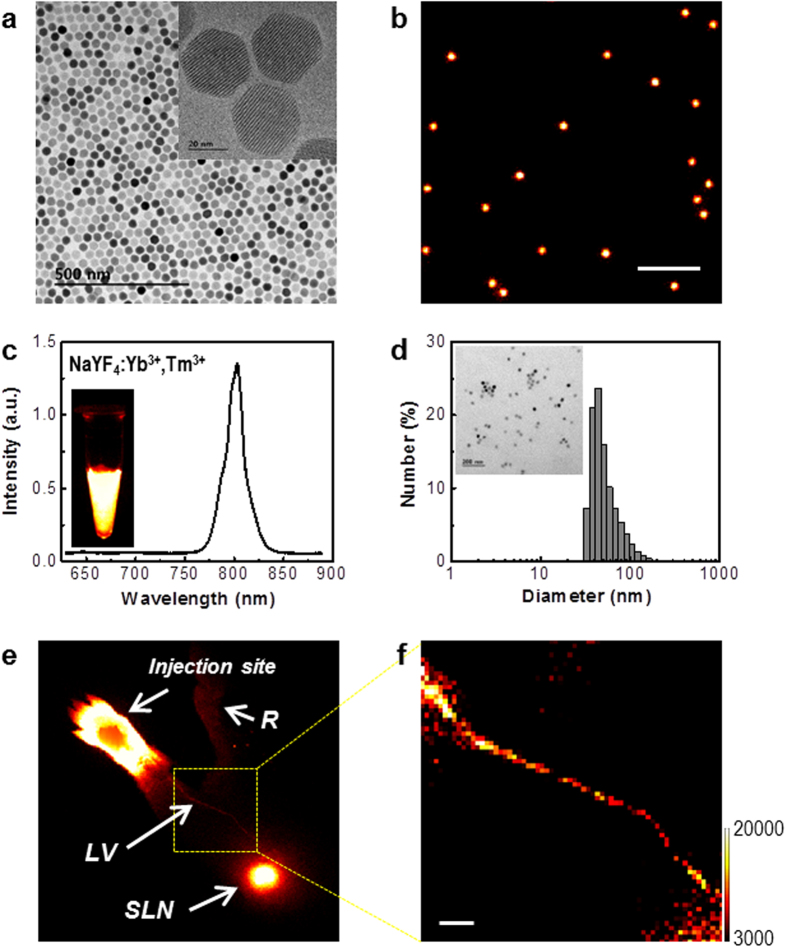
Characterization of the nanocrystals. (**a**) TEM images of NaYF_4_:Yb^3+^, Tm^3+^ nanocrystals (UCNPs). (**b**) Single particle upconversion luminescence (UCL) image of the UCNPs. The scale bar is 5 μm. (**c**) UCL spectrum of the UCNPs in organic solvent. (**d**) Size distribution of the surface-modified and water-soluble UCNPs. The inset shows TEM image of the water-soluble UCNPs. (**e**) *In vivo* UCL image of mice after injection of the surface-modified UCNPs. LV, lymphatic vessel; SLN, sentinel lymph node; R, reflected secondary emission from the injection site. (**f**) Magnified UCL image of lymphatic vessel. The scale bar is 1 mm. The size of one pixel is approximately 120 × 120 μm.

Hexagonal-phase NaYF_4_:Yb^3+^,Tm^3+^ nanocrystals were synthesized with a uniform diameter of approximately 27 nm, as shown in the high-resolution transmission electron microscope (HRTEM) image (Fig. 1a, Supplementary Fig. S1) and X-ray diffraction (XRD) pattern (Supplementary 2a). The atomic ratio (mol%) of the doped ions was determined as Y:Yb:Tm = 80.9:18.3:0.8 by inductively coupled plasma (ICP) atomic emission spectroscopy analysis. Their upconversion luminescence (UCL) emissions were evaluated at the single particle level with homogeneous and bright NIR emissions around 800 nm from each particle (Fig. 1b,c), which originated from the transition (^3^H_4_ → ^3^H_6_) of activator ions (Tm^3+^) by 980 nm excitation of sensitizer ions (Yb^3+^). These NIR-to-NIR UCL emissions provide highly effective transmissions of excitation and emission lights to deeper areas in the tissues as a biological window for *in vivo* optical imaging^16^,^17^.

To improve biocompatibility of UCNPs for *in vivo* imaging, the hydrophobic surface was modified by carboxylate functionalized poly(ethylene glycol)-lipid (PEG-lipid) (Supplementary Fig. S2b). The modified UCNPs were well dispersed in water and had a mean size of 54 nm and a surface charge of approximately −8 mV as confirmed by dynamic laser scattering (DLS) and zeta potential measurement (Fig. 1d). It was reported that the nanoparticles with a size of around 50 nm show optimal behaviour for transport and retention in lymph node mapping^18^,^19^. The negatively charged nanoparticles have advantages for *in vivo* applications because of their relatively less interaction with biological substances, such as proteins or cells with negative surface charges, in blood flow or lymphatic circulation. In particular, they provide less resistance to interstitial transport so that their lymphatic uptake is facilitated^14^,^20^. We performed *in vivo* UCL imaging of the lymphatic system in mice using the synthesized UCNPs. After injection of a UCNP solution into the forepaw footpad of the mice, UCL emission was detected using a home-built imaging system^21^, which was well designed and suitable for NIR-to-NIR *in vivo* UCL imaging, with extremely low background with minimally residual stray excitation light (Fig. 1e). The lymphatic system rapidly transported the UCNPs to SLN through lymphatic flow, and UCL emission from the SLN was clearly detected. After removal of skin, the lymphatic vessel from the injection site to the corresponding SLN was also clearly observed with bright UCL emissions, as shown in Fig. 1f.

To evaluate the SNR of regions of interest (ROIs) in the measured UCL images, two differently sized ROIs were examined as shown in Supplementary Fig. S3. The measured SNR for UCL emission of the lymph node with smaller ROIs was approximately 142 when the power density was under 200 mW/cm^2^, which is 4 times higher than those reported in previous studies^11^. In the UCL imaging of a small animal, reduction of the overheating effect with 980 nm excitation light should be considered to avoid thermal damage such as burn or fibrosis. In this study, no thermal effect on the animal was observed during UCL imaging, which enables repeated imaging with high temporal resolution in the same animal for a long period, which is consistent with the previous studies^7^. We could also confirm it by hair regrowth after few days at the depilation point for imaging.

**Figure 2:**
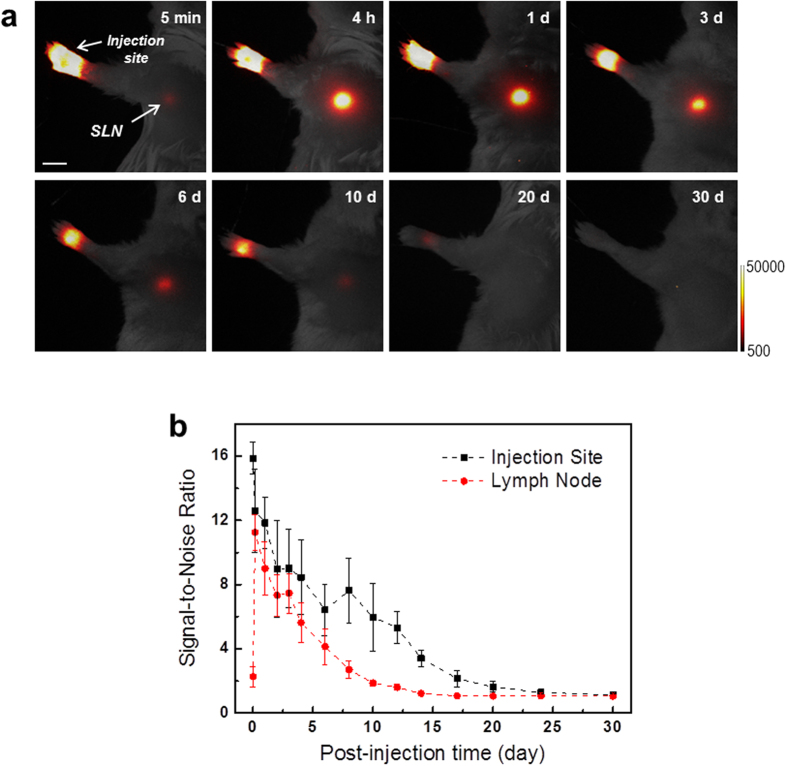
*In vivo* temporal behaviour of UCL. (**a**) *In vivo* UCL imaging of lymph node in the same mouse at different post-injection times. Mice were subcutaneously injected in the forepaw footpad with the UCNPs. The scale bar is 5 mm. (**b**) Comparison of time-dependent signal-to-noise ratio values of UCL signals between the injection site and axillary lymph node of mice (*N* = 4).

Figure 2 shows the *in vivo* temporal behaviour of UCL at the injection site and SLN after the injection. The UCL intensity in SLN indicated a maximum at 4 h post-injection and retained a relatively strong intensity for 4 days. The intensity gradually decreased in SLN as well as at the injection site and was rarely detected at 30 days after the injection. The UCL intensity dropped to approximately 2% at 14 days post-injection compared to that of 4 h post-injection (Supplementary Fig. S4). Note that the measured UCL intensity is totally attributable to the number of UCNPs remaining in the ROIs, because there was no photobleaching in the UCNPs, and thus no significant signal drop resulted from the UCNP solution for 30 days. We also performed experiment to find the effect of three different surface-functional groups, methoxy-PEG, amine-PEG, and carboxy-PEG (Supplementary Fig. S5). It was observed that the UCL intensity of UCNP functionalized with carboxy-PEG is the largest out of three cases in SLN. However, we could not find any significant difference along them in viewpoint of long-term monitoring.

**Figure 3:**
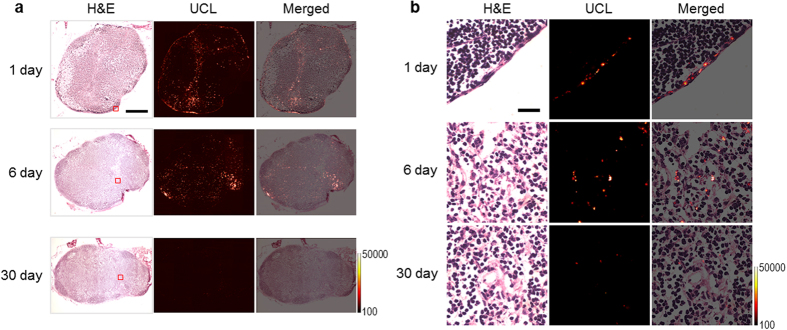
Spatial distribution of the internalized UCNPs in the lymph nodes. (**a**) H&E staining and corresponding UCL images of dissected axillary lymph nodes from the mice treated with UCNPs. The lymph nodes were obtained on 1, 6, and 30 days after the injections. The scale bar is 0.5 mm. (**b**) Magnified images of the areas marked by red rectangles in (**a**). The scale bar is 30 μm.

To further investigate the spatial distribution of the internalized UCNPs in the lymph nodes, UCL of the lymph nodes excised from the mice at several time points after the injection was analysed. Figure 3 shows the lymph node stained with haematoxylin and eosin (H&E) and the corresponding UCL images (a) and their magnified high-resolution images (b). At 1 day post-injection, the UCNPs were observed mainly in the subcapsular and trabecular sinus regions, whereas the UCNPs were rarely detected in deep cortex regions in the lymph node. This suggests that most of the UCNPs supplied from the reservoir of footpad were drained out of the lymph node around this time point and did not penetrate the cortex area containing a number of immune cells. At 6 days post-injection, the UCNPs were observed relatively in the whole area of the lymph node and were distributed in deeper regions in the lymph node. The UCL intensity at the lymph node was found to be intense for several days after injection, which may be attributed to continuous feeding from the injection site even though the UCNPs were drained out of the lymph node. At one month post-injection, the UCNPs were rarely found, which suggests that most of the UCNPs were excreted from the lymph node. Detailed UCL images of the edge and centre regions of the lymph node are shown in Supplementary Fig. S6.

**Figure 4:**
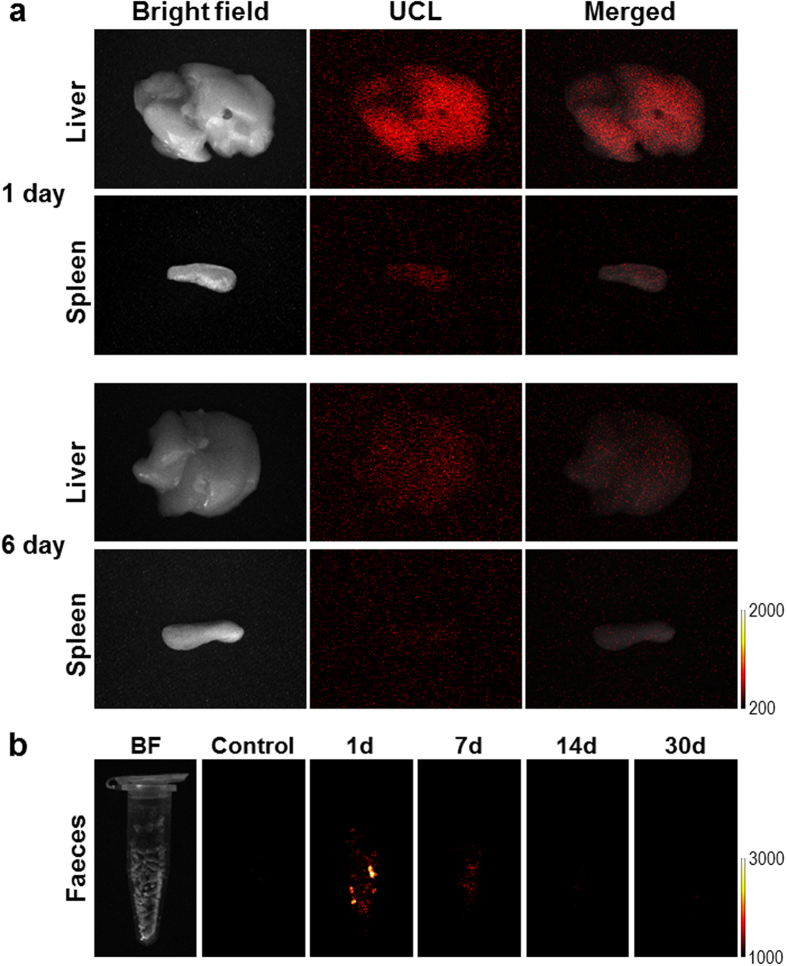
*Ex vivo* imaging of UCL from the mice. (**a**) *Ex vivo* UCL images of the major organs obtained from the mice after injection of the surface modified UCNPs at different post-injection times. (**b**) UCL images of the faeces from the mice.

To investigate the excretion pathway of the UCNPs, the mice were sacrificed at 1, 6, and 30 days after the injection of UCNPs, and the biodistribution of UCNPs in the mice were observed by UCL imaging. According to the UCL images shown in Fig. 4a, the UCNPs were found mainly in the liver and spleen at 1 day post-injection, whereas they were not detected in other organs. The signal intensity in the organs decreased at 6 days post-injection. The UCNPs were rarely detected in any organs at 30 days post-injection. Faeces from the mice were collected daily and imaged after the injection, as indicated in Fig. 4b. The UCNPs were found for the first week after the injection but no longer detected at 30 days post-injection. In accordance with the distribution changes in the lymph node, it was considered that the UCNPs were excreted by the blood circulation system through accumulation in liver and spleen following lymphatic drainage. It has been reported that molecules taken in by the lymphatic system are transported to the system circulation via the thoracic duct and subclavian veins^22^,^23^. Recently, biodistribution studies of UCNPs after intravenous injection in mice have reported clear excretion of UCNPs through the hepatobiliary pathway^21^,^24^. Here, we speculate that the subcutaneously injected UCNPs could be efficiently excreted through the hepatobiliary route in mice for up to 1 month post-injection.

In summary, we synthesized highly uniform and biocompatible Tm^3+^-doped UCNPs optimized for long-term *in vivo* imaging. It was observed that the UCNPs injected into the forepaw footpad of the mice were transported rapidly to the SLN and accumulated in the SLN for 3–4 days. According to the biodistribution the injected UCNPs were transported from the lymphatic system to the blood stream and eventually excreted in approximately 1 month post-injection. These results suggest potential applications of UCNPs with high-quality images for non-invasive, quantitative long-term *in vivo* optical imaging in the biological and medical fields, especially in biochemistry with applications in metastatic tumour detection in lymph node or in *in vivo* image-tracking of the UCNP-labelled immune cells for cell therapy.

## Methods

### Chemicals

YCl_3_·6H_2_O (99.99%), YbCl_3_·6H_2_O (99.9%), TmCl_3_ (99.9%), oleic acid (≥99%), 1-octadecene (≥95.0%), sodium hydroxide (97%), and ammonium fluoride (≥98%) were purchased from Sigma-Aldrich and used without further purification. 1,2-distearoyl-*sn*-glycero-3-phosphoethanolamine-N-[carboxy(polyethylene glycol)-2000] (DSPE-PEG-COOH) was purchased from Nanocs Inc. (New York, NY, USA).

### Synthesis of hexagonal-phase NaYF_4_:Yb^3+^,Tm^3+^ nanoparticles

Hexagonal-phase UCNPs were synthesized using previously reported methods^25^. Y-oleate (0.79 mmol), Yb-oleate (0.20 mmol), and Tm-oleate (0.01 mmol) complexes were mixed with oleic acid (8 ml) and 1-octadecene (15 ml) in a 100 ml three-neck round bottom flask under vacuum for 30 min. The mixture was heated to 100 °C under vacuum with stirring for 30 min and then cooled to room temperature under Ar atmosphere. NaOH (2.5 mmol) and NH_4_F (4 mmol) dissolved in methanol (10 ml) were slowly added to the reaction mixture under Ar atmosphere at 50 °C. The mixture was heated to 100 °C under vacuum with stirring for 30 min until residual methanol was completely removed. Subsequently, it was heated to 300 °C at a constant heating rate of 3.3 °C/min and kept at that temperature for 1.5 h under Ar atmosphere. After cooling down to room temperature, UCNPs were precipitated by adding excess ethanol and isolated by centrifugation performed 3 times. The purified UCNPs were dispersed in hexane.

### Surface modification of nanoparticles by carboxylic acid functionalized phospholipid-PEG

1 mg of UCNPs was dispersed in 100 μl of chloroform. 10 mg of DSPE-PEG-COOH was dispersed in water in a sonication bath for 30 min. UCNP solution was slowly added to lipid solution during tip sonication (Branson Sonifier, Danbury, USA). The emulsion was vigorously vortexed for 5 min and stirred until residual chloroform was completely removed. The surface-modified UCNPs were purified by centrifugation and dispersed in water.

### Characterization of nanoparticles

The synthesized nanoparticles were characterized by HRTEM and XRD. TEM images were obtained on a Philips Tecnai F20 electron microscope, and the XRD pattern was produced on a Bruker AXS (D8 DISCOVER) instrument equipped with Cu K_α_ radiation (λ = 1.5418 Å). Particle size distribution and surface zeta potential were analysed on a Zetasizer Nano ZS instrument (Malvern Instruments, Malvern, UK). Elemental analysis of nanoparticles was performed by ICP optical emission spectroscopy (Optima 8300DV or 4300DV, Perkin Elmer, Waltham, MA, USA).

### Measurement of the upconversion luminescence spectra and images of UCNPs

The UCNP solutions were excited by a 980 nm continuous-wave (CW) single-mode diode laser (P161-600-980A, EM4, Andor Technology, Belfast, UK). Their emission was collected through an optical fibre by a home-made UCL spectrograph and imaging system, which was composed of an inverted microscope (IX71, Olympus, Tokyo, Japan) and an electron multiplying charge coupled device (EMCCD) camera (DV897DCS-BV, iXon, Andor Technology, Belfast, UK). It was detected by a CCD camera (PIXIS 400BR, Princeton Instruments, Trenton, NJ, USA) attached to an imaging spectrograph (IsoPlane SCT320, Princeton Instruments). The images of individual UCNPs or UCNPs in sliced SLN tissues were taken from the same imaging system. To obtain colour images of SLN tissues, a colour sCMOS camera (OS4MPc-CL-RGB, Raptor Photonics, Larne, UK) was employed.

### *In vivo* and *ex vivo* UCL imaging

Male BALB/c mice (6 weeks of age) were purchased from OrientBio (Kyunggi-do, Korea), and the imaging areas were treated with a depilatory cream. Surface-modified UCNP solution (20 μl of 25 pM) was injected subcutaneously into the forepaw footpad. To supply homogeneous nanoparticles to the animals, we injected the particles right after removing the aggregates by syringe filtration with a correction of its concentration. Then the mice (n = 4) were placed in a home-made high-quality UCL *in vivo* imaging system connected to an EMCCD camera (DU897-EX, iXon Ultra, Andor Technology). UCL images of mice were acquired using a high-power (0–30 W) 980 nm diode laser (Dilas, Lysyn Yepli, Bucheon, Korea) as an excitation light source and two emission filters (FF01-800/12 and FF01-890/SP, Semrock, Rochester, NY, USA). All images were processed with the Andor Solis software (Andor Technology, Belfast, UK).

This *in vivo* study followed the guidelines of the committee on animal research at the Korea Basic Science Institute (KBSI), with the protocol approved by the local institutional review committee on animal care (KBSI-AEC1508). For the imaging of organs and faeces, the mice (n = 3) were sacrificed at different post-injection times and the organs (axillary lymph node, heart, lungs, kidneys, liver, stomach, spleen, intestine, and injection site) were removed and imaged using the UCL imaging system. The faeces were collected daily for the first 7 days and at 10, 14, 22, and 30 days after injection. The mice were housed in a metabolic cage to collect faeces.

### Histologic analysis

To analyse *in situ* distribution of UCNPs, three groups of the mice (n = 3 per group) were sacrificed on 1, 6 and 30 days, and then axillary lymph nodes were dissected and embedded in a Tissue-Tek OCT compound (Sakura, Tokyo, Japan) and frozen in liquid nitrogen. Cryosections (~8 μm) were prepared by a cryostatic microtome (Leica Microsystems Nussloch GmbH, Nussloch, Germany) and were transferred to glass slides and stained with hematoxylin and eosin (H&E).

## Additional Information

**How to cite this article**: Park, H. S. *et al.* Clear-cut observation of clearance of sustainable upconverting nanoparticles from lymphatic system of small living mice. *Sci. Rep.* **6**, 27407; doi: 10.1038/srep27407 (2016).

## Supplementary Material

Supplementary Information
