# Supplementary material for: Clear-cut observation of clearance of sustainable upconverting nanoparticles from lymphatic system of small living mice
Source: Sci Rep. 2016 Jun 6;6:27407. doi: 10.1038/srep27407 (PMC4893699; doi:10.1038/srep27407)
Supplement: Supplementary Information [file srep27407-s1.doc]

**Supplementary Information for
Clear-cut observation of clearance of sustainable upconverting nanoparticles from lymphatic system of small living mice**

Hye Sun Park^1,†^, Sang Hwan Nam^2,†^, Jongwoo Kim^2^, Hyung Seon Shin^3^, Yung Doug Suh^2,4,∗^ & Kwan Soo Hong^1,5,6,∗^

1. Bioimaging Research Team, Korea Basic Science Institute, Cheongju 28119, Korea
2. Laboratory for Advanced Molecular Probing (LAMP), Research Center for Convergence NanoRaman Technology, Korea Research Institute of Chemical Technology, Daejeon 34114, Korea
3. Environmental Monitoring & Research Team, Korea Basic Science Institute, Cheongju 28119, Korea
4. School of Chemical Engineering, Sungkyunkwan University, Suwon 16419, Korea
5. Bioanalytical Science, University of Science and Technology, Daejeon 34113, Korea
6. Graduate School of Analytical Science and Technology, Chungnam National University, Daejeon 34134, Korea

**Supplementary Figure S1 |** **Characterization of the UCNPs.** (**a**) TEM Image of NaYF_4_:Yb^3+^,Tm^3+^ nanocrystals (UCNPs) dispersed in hexane. (**b**) Magnified TEM image of the UCNP. (**c**) Dynamic light scattering (DLS) histogram for UCNPs dispersed in hexane. The average diameter is 27.6 ± 6.9 nm.

**Supplementary Figure S2 |** **Characterization of the UCNPs and their surface-modified ones.** (**a**) X-ray diffraction pattern of the UCNPs (upper). All diffraction peaks match well with the standard pattern of hexagonal NaYF_4_ (JCPDS file number 28-1192) (lower). (**b**) FT-IR spectra of i) the as-prepared UCNP, ii) DSPE-PEG-COOH and iii) the modified (carboxylate functionalized) UCNP. The transmission bands at 2917 and 2851 cm^-1^ in i) and iii) are respectively assigned to the asymmetric (*ν*_as_) and symmetric (*ν*_s_) stretching vibrations of methylene (CH_2_) in the long alkyl chain. A broad band at around 3400 cm^-1^ in ii) and iii) attribute to the stretching vibration of O-H in carboxyl group of PEG-COOH. A peak at 1106 cm^-1^ in ii) and iii) attributed to the –C-O-C- group of PEG on the surface of UCNPs.

**Supplementary Figure S3 |** **Quantitative analysis of photoluminescence signals of the axially lymph node at different ROIs for *in vivo* animal imaging.** (**a**) *In vivo* UCL images of the mouse after injection of the UCNPs with 6 representative ROIs (yellow boxes; ROI1 to ROI6). (**b**) and (**c**) Comparison of SNR (or contrast-to-noise, CNR) calculated from measured intensities between two different cases of ROI groups; larger CNR values are obtained for smaller ROIs (**c**), which have 1/4-fold number of pixels of (**b**).


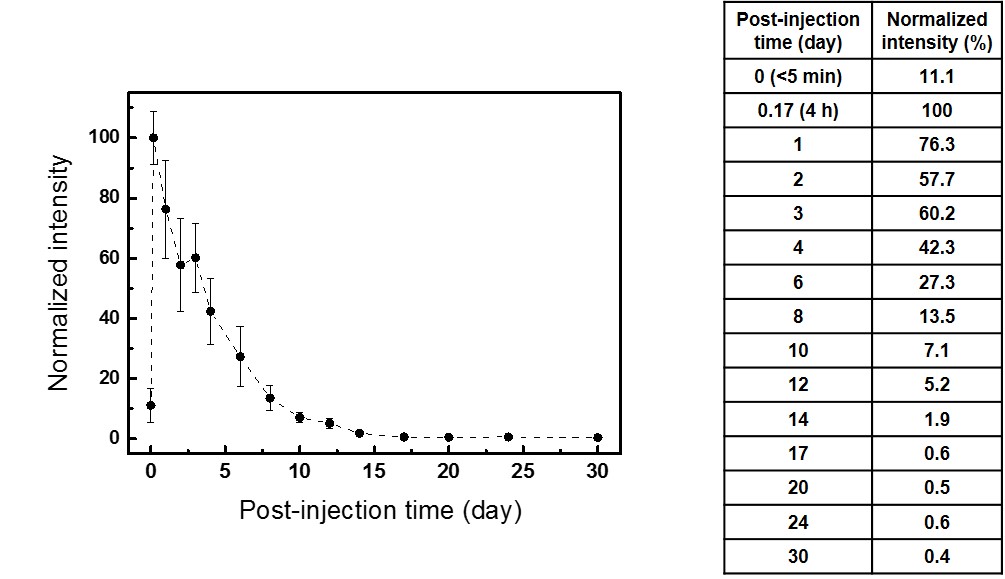


**Supplementary Figure S4 |** ***In vivo* temporal behaviour of UCL.** Normalized UCL intensities for the lymph node at different post-injection times.

**Supplementary Figure S5 |** **The effect of three different surface-functional groups.** (a) UCL images, (b) size distributions and surface charges of three different surface-functionalized (modified by methoxy-PEG, amine-PEG, and carboxy-PEG) UCNPs. Comparison of time-dependent signal-to-noise ratio values of UCL among the three different surface-functionalized UCNPs in (c) axillary lymph node and (d) injection site.

**Supplementary Figure S6 |** **Spatial distribution of the internalized UCNPs in the lymph nodes.** (**a**) H&E staining and corresponding UCL images of dissected axillary lymph nodes from the mice treated with UCNPs. The lymph nodes were dissected at 1, 6, and 30 days after the UCNP injection. The areas marked by red rectangles in (**a**) are correspondingly shown in (**b**) (edge regions) and (**c**) (centred regions of the lymph nodes) as magnified images. The scale bars in (**a**) and (**b**) are 0.5 mm and 30 μm, respectively.
